# SnS@C nanoparticles anchored on graphene oxide as high-performance anode materials for lithium-ion batteries

**DOI:** 10.3389/fchem.2022.1105997

**Published:** 2023-01-04

**Authors:** Jing Mei, Jinlu Han, Fujun Wu, Qichang Pan, Fenghua Zheng, Juantao Jiang, Youguo Huang, Hongqiang Wang, Kui Liu, Qingyu Li

**Affiliations:** ^1^ Guangxi Key Laboratory of Low Carbon Energy Materials, School of Chemistry and Pharmaceutical Sciences, Guangxi Normal University, Guilin, China; ^2^ Guangxi New Energy Ship Battery Engineering Technology Research Center, Guangxi Normal University, Guilin, China

**Keywords:** SNS, N-doped carbon, graphene oxide, anode, lithium-ion batteries

## Abstract

Tin (II) sulfide (SnS) has been regarded as an attractive anode material for lithium-ion batteries (LIBs) owing to its high theoretical capacity. However, sulfide undergoes significant volume change during lithiation/delithiation, leading to rapid capacity degradation, which severely hinders its further practical application in lithium-ion batteries. Here, we report a simple and effective method for the synthesis of SnS@C/G composites, where SnS@C nanoparticles are strongly coupled onto the graphene oxide nanosheets through dopamine-derived carbon species. In such a designed architecture, the SnS@C/G composites show various advantages including buffering the volume expansion of Sn, suppressing the coarsening of Sn, and dissolving Li_2_S during the cyclic lithiation/delithiation process by graphene oxide and N-doped carbon. As a result, the SnS@C/G composite exhibits outstanding rate performance as an anode material for lithium-ion batteries with a capacity of up to 434 mAh g^−1^ at a current density of 5.0 A g^−1^ and excellent cycle stability with a capacity retention of 839 mAh g^−1^ at 1.0 A g^−1^ after 450 cycles.

## 1 Introduction

Lithium-ion batteries (LIBs) have been widely used in portable electronic devices, energy storage systems, and hybrid/electric vehicles because of their high energy density, excellent safety performance, light weight, and environmental friendliness (Simon and Gogotsi, 2008; Kang and Ceder, 2009; Ji et al., 2011). With the rapid development of the burgeoning market of electric vehicles, the energy density of present lithium-ion batteries has been unable to meet the requirement of long driving range, and the exploration of high energy density LIBs has become an urgent need. However, the mainstream commercial anode electrode materials of graphite suffer from a theoretical specific capacity of 372 mAh g^−1^, which limits the further improvement of the energy density of LIBs (Choi et al., 2012; Ding et al., 2022a). Therefore, developing high-capacity anode electrode materials becomes the essential strategy to achieve high energy density LIBs (Shen et al., 2018; Cui et al., 2020; Ding et al., 2022b; Zhao et al., 2022). Up to now, a large number of high-capacity anode electrode materials, including alloy-based anodes, metal oxides, and metal sulfides, are being investigated as promising anode materials for the next-generation of high energy density LIBs owing to their high capacity (Li et al., 2017; Pan et al., 2018; Pan et al., 2020).

Among these potential anode materials, metal sulfides, such as FeS, SnS, NiS, and ZnS, are receiving considerable scholarly attention due to their abundant reserve and high capacity (Kim et al., 2007; Seo et al., 2008; Zhang et al., 2012; Zhang et al., 2014; Wang et al., 2015). Layered tin (II) sulfide with a theoretical capacity of up to 782 mAh g^−1^ is considered to be a representative anode material of metal sulfides for LIBs (Tripathi and Mitra, 2014; Chauhan et al., 2015; Li et al., 2015; Pan et al., 2017). Meanwhile, the large layer spacing of SnS (.433 nm) can also provide a pathway to facilitate the insertion/de-insertion of lithium ions (Nassary, 2005; Liu et al., 2016; Tan et al., 2016; Huang et al., 2021). However, SnS, as an anode material, suffers from significant volume change during lithiation/delithiation, resulting in rapid capacity degradation and poor electrical conductivity. As a result, the practical application of SnS in LIBs is seriously hindered (Zhang et al., 2012; Li et al., 2015; He et al., 2017). To address the issue, various methods have been tried, including nanostructure design and carbon hybridization, which have been proven to be effective in improving the performance of SnS anode (Li et al., 2007; Liu et al., 2014; Lian et al., 2017; Xue et al., 2018). Therein, carbon hybridization has been consistently recognized as the most effective method in previous studies, as the carbonaceous material can buffer the volume change of SnS during the lithiation/delithiation process and also improve the electrical conductivity of the mixture (Zhang et al., 2012; Leung et al., 2013; Lu et al., 2015). Therefore, various SnS-based composites combined with carbonaceous material have been developed as anode materials for LIBs and show the ability to significantly improve the electrochemical performance (Pol et al., 2008; Liu et al., 2016). For example, Ma et al. (2019b) reported that SnS/graphene nanosheets with N-carbon as the outer coating layer could effectively inhibit the volume expansion of SnS particles and improve the conductivity, resulting in a significant improvement in electrochemical performance. Xue et al. (2018) prepared a 3D interconnected macroporous carbon material using silicaopal as a template, which can limit the volume expansion of SnS nanoparticles, improve the electrical conductivity, and maintain the structural integrity. Zhao et al. (2017b) self-assembled SnS nanoparticles from graphene oxide nanosheets and positively charged polystyrene/tin dioxide nanospheres, and SnS nanoparticles were tightly and uniformly immobilized on the graphene surface with excellent properties. While, for these mentioned above SnS-based anode materials, which usually hardly to achieve long cycle performance over 400 cycles, the reason can be ascribed to the carbonaceous material have poor adhesion with SnS anode. Then, during the cycling process, the SnS will be divorced from the carbonaceous material, resulting in continuous capacity fading and poor cycle performance.

In this work, we designed and synthesized SnS@C/G composites through *in situ* complexation and polymerization guided by graphene oxide as a structure-orientating agent followed by *in situ* sulfuration treatment for curing. In such an attractive structure, the SnS@C nanoparticles are strongly coupled onto graphene oxide nanosheets *via* the carbon species derived from dopamine (PDA), offering various advantages as follows: 1) The amorphous N-doped carbon layer increases the electrical conductivity of the composite and also prevents the direct contact between the electrolyte and internal SnS, which contributes to the formation of a stable SEI film; 2) The N-doped carbon layer buffers the volume change of SnS during cycling, while inhibiting the coarsening of Sn and the dissolution of sulfur; 3) Graphene oxide nanosheets can effectively improve the electrical conductivity of the composite while buffering the volume change of SnS during the discharge/charging process.

## 2 Experimental

### 2.1 Preparation of graphene oxide

Graphene oxide was synthesized by electrochemical exfoliation method with graphite powder as raw material. Briefly, graphite powder was encapsulated in a nylon fiber bag as the anode and a titanium sheet was employed as the cathode in a dual-electrode system with an inter-electrode distance of 2 cm. The electrochemical exfoliation was carried out at a DC voltage of 10 V for 4 h with .1 M sodium sulfate as electrolyte. The final electrochemically exfoliated graphene oxide was obtained through successive post-treatments of the exfoliated product including wash, sonication, centrifugation at 3,000 rpm, and vacuum freeze-drying.

### 2.2 Preparation of SnS@C/G composite

To synthesize the SnS@C/G composite, .266 g of sodium stannate was first added to a solution with 80 ml of deionized water and 40 ml of ethanol and then stirred until completely dissolved. Subsequently, graphene oxide was added and sonicated for 30 min. After that, .2 g of PDA and 1.0 ml of ammonia water were added, and the resulting mixture was stirred continuously for 12 h. Then, the mixture was separated by centrifugation at 8,000 rpm and the obtained product was washed three times with deionized water and dried at 80°C for 12 h. Finally, the SnS@C/G materials were obtained by annealing with thiourea at 600°C for 2 h under nitrogen atmosphere. As a comparison, SnS@C was also prepared without graphene oxide under the same conditions.

### 2.3 Characterizations

The morphology and structure of samples were observed by SEM (SIGMA 300, ZEISS), TEM (Talos 200S, ThermoFisher Scientific), and HRTEM. The crystal structure information and composition were investigated using XRD (D/Max-3c, Rigaku). The elemental composition and the surface valence state were analyzed using XPS (NEXSA X, ThermoFisher Scientific). The thermogravimetric analyses (TGA) were carried out (STA 449F3, Netzsch) from 25°C to 1,000 °C at a heating rate of 10 °C min^−1^ under air. Raman characterization (inVia Quotation, Renishaw) was conducted in the air for radiation at 735 nm.

### 2.4 Electrochemical measurements

A CR2025-type cell for electrochemical measurements was prepared with electrode consisting of 80% of the prepared material, 10% carbon black, and 10% carboxymethyl cellulose (CMC). The mass loading of active materials is .72 mg cm^−2^1 M LiPF_6_ dissolved in ethylene carbonate (EC)/diethyl carbonate (DEC) (1:1, *v*) with 5% fluoroethylene carbonate (FEC) was used as the electrolyte. Cyclic voltammetry was conducted between .01 and 3.0 V with different scan rates from .1 to 2.0 mV s^−1^. EIS was recorded over a frequency range of .01–10^5^ Hz. The discharge/charge measurement was performed with a voltage range of .01–3.0 V on the Land battery test system.

## 3 Results and discussion

**FIGURE 1:**
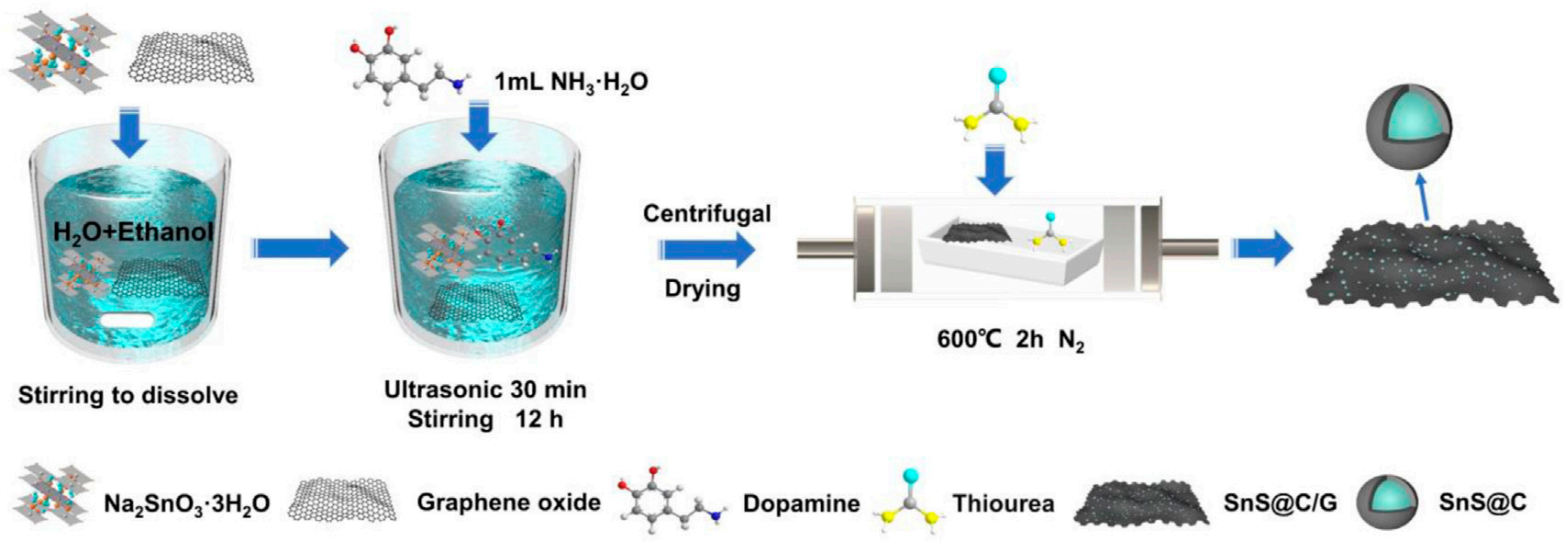
Schematic diagram of the preparation process of SnS@C/G composite.

The preparation process of the SnS@C/G composite is illustrated in Figure 1. First, during the dissolution or dispersion of sodium stannate, graphene oxide, and PDA by ultrasonic treatment, SnO_3_^2-^ binds to PDA *via* a complexation reaction. Then, PDA undergoes an *in situ* polymerization reaction on the surface of graphene oxide nanosheets. Finally, the SnS@C/G composite was synthesized by freeze-drying and *in situ* sulfuration. All these diffraction peaks in the XRD patterns of SnS@C/G and SnS@C shown in Supplementary Figure S1 correspond to the peaks of the standard card of SnS (JCPDS No. 39–0354), indicating the successful preparation of high-purity SnS-based materials (Wu et al., 2014). The defect and structural integrity of the carbon structure was further investigated by Raman and the corresponding spectra are shown in Supplementary Figure S2. It is observed the characteristic peaks of the disordered and ordered sp^2^ bonds are located at 1,311 and 1,575 cm^−1^, respectively (Ferrari and Robertson, 2000). Meanwhile, the *I*_*D*_/*I*_*G*_ value of the SnS@C/G composite (1.78) is larger than that of the SnS@C composite (1.46), which is mainly attributed to the presence of amorphous carbon in the SnS@C/G composite (Fang et al., 2013; Zhu et al., 2015). In addition, due to the presence of graphene oxide and amorphous carbon, the weight of the SnS@C/G composite decreased significantly by 40% during the thermogravimetric analysis in the air from 100°C to 900°C as shown in Supplementary Figure S3. Therefore, the content of SnS is determined to be 60% in the SnS@C/G composite according to the TGA result.

**FIGURE 2:**
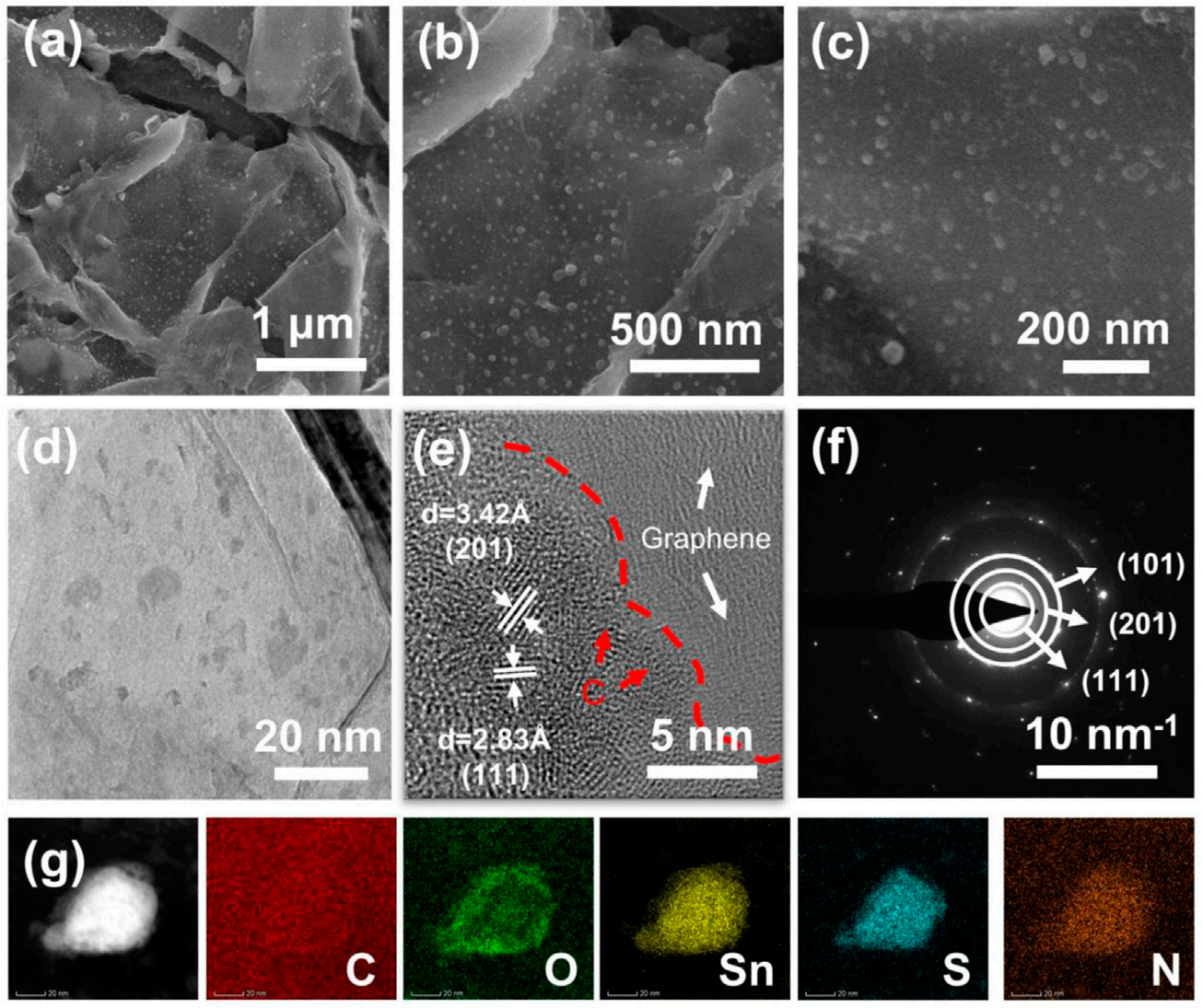
**(A–C)** SEM, **(D)** TEM, **(E)** HRTEM, **(F)** SAED, and **(G)** STEM images and corresponding elemental mappings of C, O, Sn, S, and N of SnS@C/G composite.

The surface morphology and microstructure of SnS@C/G composites were observed by SEM. It can be observed that the SnS@C/G composites have a typical sheet-shaped structure as shown in Figure 2A. The corresponding HRSEM images as shown in Figures 2B, C further show that many ultra-small SnS nanoparticles are embedded in nanosheets with particle size less than 10 nm. As a comparison, the SnS@C composite exhibits a bulk structure observed from the SEM images as shown in Supplementary Figure S4, and the TEM images demonstrate that a carbon coating layer on the surface of SnS particles (Supplementary Figure S5). To further observe the microstructure of the SnS@C/G composite, the TEM measurement was carried out. The TEM image shown in Figure 2D further confirms the sheet-shaped structure and demonstrates that many nanoparticles are embedded in nanosheets. Furthermore, the HRTEM image shown in Figure 2E reveals that the lattice spacings of the nanoparticles are 3.42 Å and 2.83 Å, corresponding to the (201) and (111) planes of SnS. Moreover, an amorphous carbon layer can be seen on the surface of the SnS particles, demonstrating that the SnS nanoparticles are coated by an N-doped carbon layer derived from PDA. In addition, it can still be observed that the SnS@C nanoparticles are embedded in the graphene oxide nanosheets. Figure 2F further shows that the SAED image of SnS@C/G nanoparticles, and the resulting diffraction rings in the figure match well with the (111), (201), and (101) planes of the orthogonal SnS phase. Finally, the elemental composition and distribution of SnS@C/G were analyzed using STEM and EDS. As shown in Figure 2G, it is observed that the elements of C, O, Sn, S, and N are uniformly distributed on the SnS@C/G nanoparticles.

**FIGURE 3:**
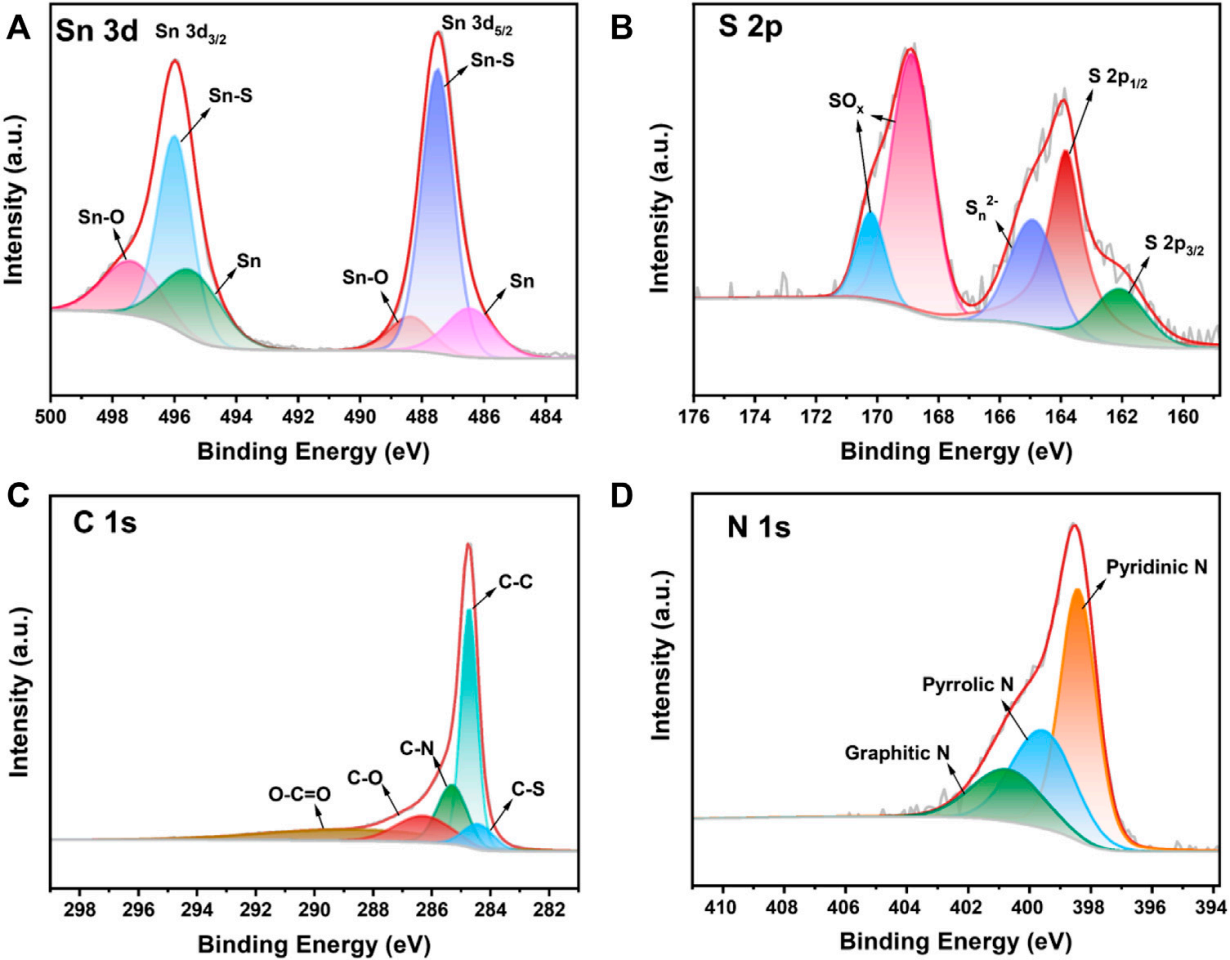
High-resolution XPS spectra of the SnS@C/G composite: **(A)** Sn 3d, **(B)** S 2p, **(C)** C1s, and **(D)** N 1s.

Elemental composition and elemental valence states of SnS@C/G composites are then analyzed by XPS. The survey scan of the SnS@C/G composite was conducted and the spectrum is shown in Supplementary Figure S6, which reveals the presence of Sn, S, C, N, and O elements. In the high-resolution spectrum of Sn 3d shown in Figure 3A, two peaks at 487.5 and 496.0 eV assigned to Sn 3d_5/2_ and Sn 3d_3/2_, respectively, can be observed (Zhu et al., 2015; Zhao et al., 2016), demonstrating the presence of Sn^2+^ derived from SnS. Besides, the peaks at 497.4 and 488.3 eV can be attributed to Sn-O, which may be due to the oxidation of SnS in the air. In the high-resolution spectrum of S 2p as shown in Figure 3B, the peaks at the binding energies of 162.0 and 163.9 eV correspond to S 2p_3/2_ and S 2p_1/2_ (Ren et al., 2012). Two peaks at 168.9 and 170.2 eV can be observed due to the partial oxidation of sulfur at the surface in the air, resulting in the formation of SO_x_ (Zhou et al., 2020). Moreover, in the high-resolution spectrum of C 1s as shown in Figure 3C, there are five peaks centered at 284.4, 284.8, 285.3, 286.3, and 288.7 eV, which can be ascribed to C-S, C-C, C-N, C=O and O-C=O (Liu et al., 2014; Wang et al., 2018b). The presence of C-N and C-S bonds again effectively demonstrates the successfully doping of N and S elements into the carbon materials. Furthermore, for the N element, the binding energies at 398.4, 399.6, and 400.8 eV correspond to the pyridyl N, pyrrolyl N, and graphitic N, respectively, as shown in Figure 3D (Xiong et al., 2017; Xia et al., 2019).

**FIGURE 4:**
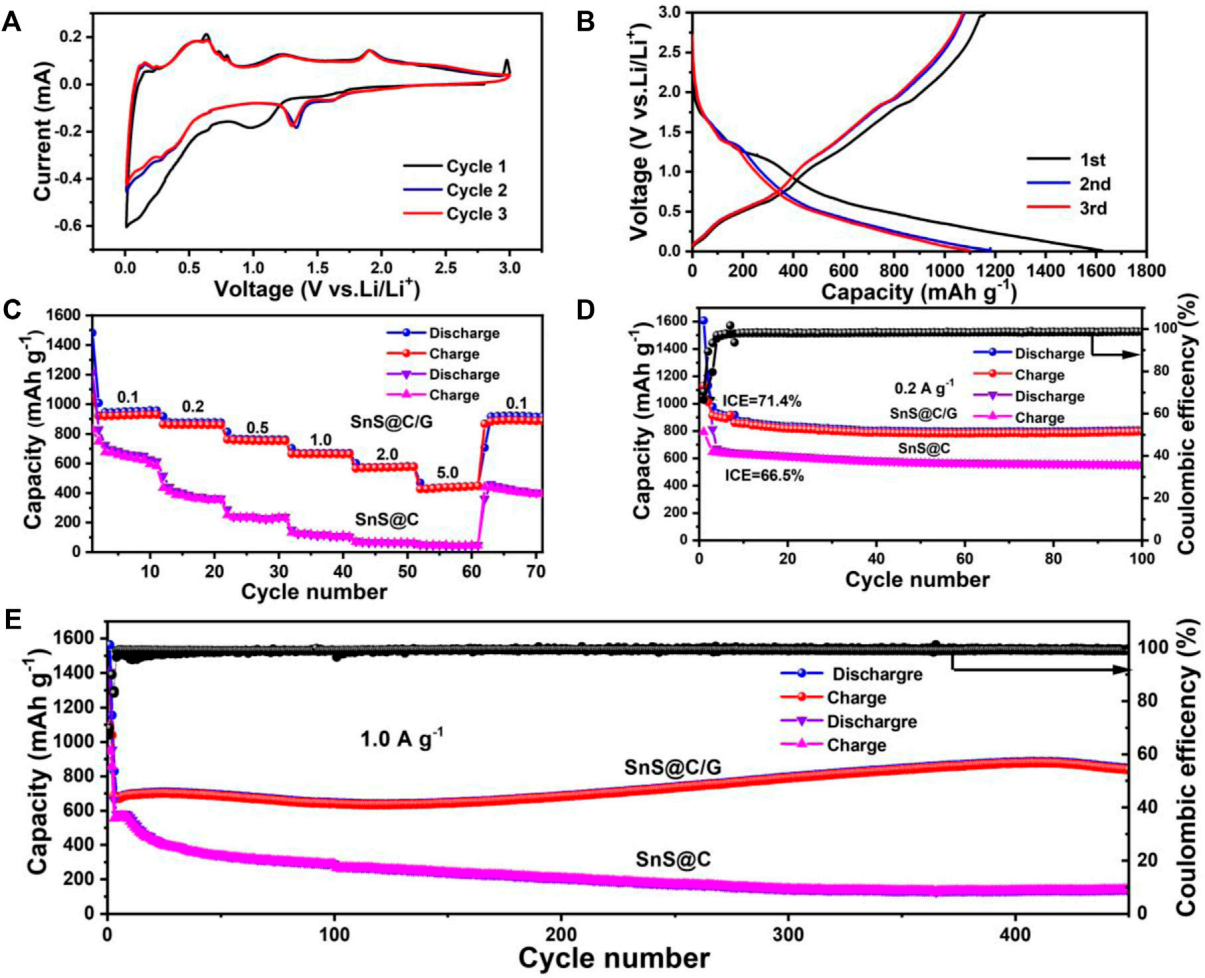
Electrochemical performances of composites for lithium batteries: **(A)** CV curves at a scanning rate of .1 mV s^−1^ and **(B)** charge and discharge voltage profiles of the SnS@C/G composite for the first three cycles at 0.1A g^−1^; **(C)** Rate performances, **(D)** cycling performance at .2 A g^−1^, and **(E)** long-term cycling performance at 1.0 A g^−1^ of SnS@C/G and SnS@C composites.

The lithium storage behaviors of the SnS@C/G composite were further evaluated *via* Li half cells with a potential of .01–3.0 V. The CV curves for the first three cycles of SnS@C/G at .1 mV s^−1^ are shown in Figure 4A. A broad cathodic peak at .98 V appears in the first cycle, which corresponds to the conversion of SnS to Sn and Li_2_S by reaction with Li^+^ and the formation of a solid electrolyte interface (SEI) (Zhao et al., 2017a; Cheng et al., 2021). The peaks ranging from .01 to .65 V are due to the alloying of Sn with Li^+^ to Li_x_Sn alloy and Li^+^ intercalation into graphene oxide (Xu et al., 2013; Xue et al., 2018). During the anodic scan, the peaks representing dealloying as well as Li^+^ deintercalation from graphene oxide layers appear in the low potential range (<1.0 V), and the peaks of the reverse conversion reaction representing the regenerated SnS phase occur in the high potential range (>1.0 V) (Zhang et al., 2012; Gao et al., 2019; Cheng et al., 2021). After the initial cycle, the 2nd and 3rd CV curves are well overlapped, indicating the excellent reversibility of SnS@C/G composite during the cycling. By analyzing the voltage plateaus in the discharge/charge curves of the SnS@C/G composite for the first three cycles shown in Figure 4B, it can be seen that the voltage plateaus are consistent with the results of CV analysis. As a comparison, the CV curve of the SnS@C composite is shown in Supplementary Figure S7A, which shows similar shapes to the SnS@C/G composite. In addition, another peak appears around 2.3 V, which may be due to the reversible conversion of Li_2_S with polysulfide. Furthermore, the SnS@C/G composite has a higher capacity of 1,033 mAh g^−1^ than the SnS@C composite with a capacity of 791 mAh g^−1^ and also exhibits a higher initial Coulombic efficiency (ICE) of 71.4% than the SnS@C composite with an ICE of 66.5% (Supplementary Figure S7B).

The rate capability of half-cell was evaluated from .1 to 5.0 A g^−1^ and the results are shown in Figure 4C. Remarkably, the capacities of SnS@C/G composites can be maintained at 930, 863, 758, 666, 564, and 434 mAh g^−1^ when the current densities are set to .1, .2, .5, 1.0, 2.0, and 5.0 A g^−1^, respectively. When the current density returns to .1 A g^−1^, the capacity of SnS@C/G recovers to 918 mA h g^−1^ and remains essentially constant. Nevertheless, SnS@C exhibits much lower capacities of 620, 353, 226, 104, and 64 mAh g^−1^ at .1, .2, .5, 1.0, and 2.0 A g^−1^, respectively. Especially, a low capacity of 43.7 mAh g^−1^ is observed at the current density of 5.0 A g^−1^, and only a capacity of 393 mAh g^−1^ is retained when the current density returns to .1 A g^−1^. Therefore, it can be concluded that the SnS@C/G composite has a significant advantage in terms of rate performance.

The cycling properties of SnS@C/G and SnS@C composites are shown in Figures 4D, E. It can be seen that SnS@C/G exhibits outstanding cycling performance and it can still provide a high capacity of 800 mAh g^−1^ after 100 cycles at .2 A g^−1^. On the contrary, SnS@C shows relatively poor performance of only 547 mAh g^−1^ after 100 cycles. Therefore, the SnS@C/G composite exhibits better cycling performance than the SnS@C composite. In addition, the long-term cycling performance was further demonstrated at a higher current density of 1.0 A g^−1^. As shown in Figure 4E, the SnS@C/G composite still shows good long-term cycling performance; the reversible capacity can reach 839 mAh g^−1^ after 450 cycles. The increase in capacity during the cycling is probably attributed to the formation of a gel-like polymer resulting from electrolyte degradation and the activation of the electrode materials through the SEI reconstruction process (Zhang et al., 2015; Wang et al., 2018a; Sun et al., 2018; Zhang et al., 2018; Ma et al., 2019a). However, the cycling performance of the SnS@C composite is relatively very poor with a continuous capacity fading and a final capacity of only 40 mAh g^−1^ is maintained after 450 cycles. For comparison, the electrochemical performance of graphene oxide was also tested as shown in Supplementary Figure S8. As can be seen, the cycling performance and rate capability of graphene oxide are much lower than those of SnS@C/G composite. Finally, the lithium storage performance of prepared SnS@C/G composite is further compared with those of recently reported SnS-based composites for LIBs as shown in Supplementary Table S1, which clearly demonstrates the superiority of our SnS@C/G composite in terms of cycling performance and rate capability.

To better understand the outstanding lithium storage performance of the SnS@C/G composite, EIS measurements before and after 100 cycles were performed on both SnS@C/G and SnS@C composites as shown in Supplementary Figure S9. It is observed that the semicircle diameter of SnS@C/G is much smaller than that of SnS@C/G as shown in the Nyquist diagrams in Supplementary Figure S9A, indicating that the addition of graphene oxide in the SnS@C/G composite can significantly enhance its conductivity. The Li-ions diffusion rate of SnS@C/G is larger than that of SnS@C, according to the larger slope of SnS@C/G than that of SnS@C in the low-frequency region of the impedance line. After 100 cycles, the semicircular diameters of both SnS@C/G and SnS@C composites become smaller as shown in Supplementary Figures S9C, D, which may be due to the activation of the electrode materials after multiple cycling processes. Meanwhile, the Li-ions diffusion is enhanced for the SnS@C/G composite but decreased for the SnS@C composite after 100 cycles. This further indicates that SnS@C/G is capable to maintain the structural stability during cycling, thus maintaining good electrical conductivity and ionic conductivity with excellent rate performance (Zheng et al., 2016; Cheng et al., 2019). Moreover, the high conductivity of the SnS@C/G composite also can be further confirmed by the GITT results as shown in Supplementary Figure S10.

**FIGURE 5:**
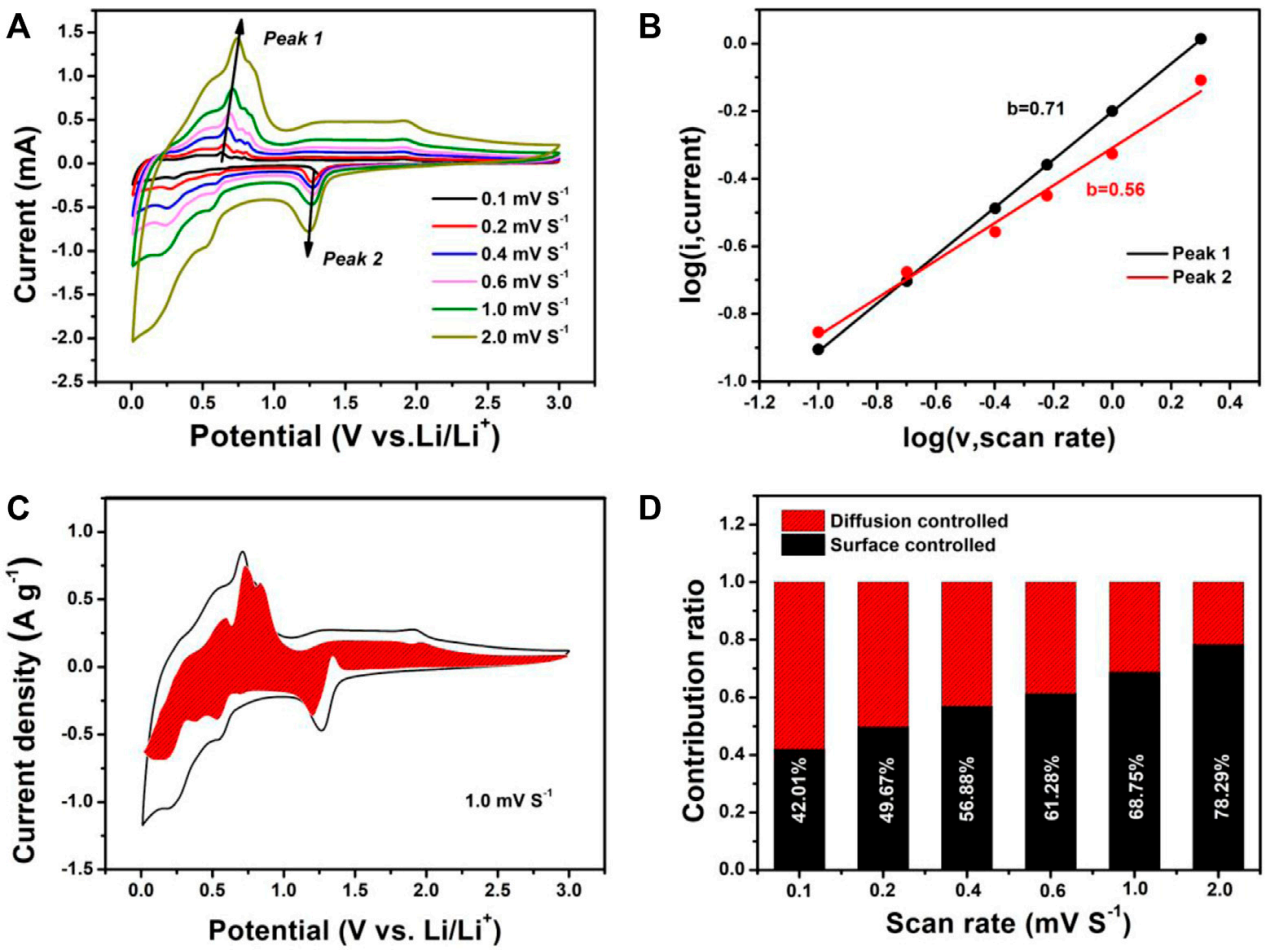
Electrochemical kinetics of the SnS@C/G composite for lithium storage: **(A)** CV curves at various scan rates, **(B)** determination of the b value using Eq. 1, **(C)** normalized contribution ratio of the capacitive and diffusion current at a scan rate of 1.0 mV s^−1^, **(D)** contribution of the capacitive and diffusion-controlled charge transport process at different scan rates.

To further understand the excellent rate performance of the SnS@C/G composite, the electrochemical kinetics were investigated by the CV test at various scan rates from .1 to 2.0 mV s^−1^, and the corresponding CV curves are shown in Figure 5A. It can be seen that all CV curves are similar in shape, indicating a slight polarization in the SnS@C/G composite. According to Eq. 1, the mathematical relationship between the current (*i*) and the scanning speed (*v*) is obtained (Lindstrom et al., 1996):

$$i=av^{b}$$ (1)

where a and b are adjustable parameters obtained by logarithmic operations on the current over the scan rate. The application of Eq. 1 to the SnS@C/G composite is shown in Figure 5B. It can be calculated that the b value of peak 1, the anode peak, is .71 and the b value of peak 2, the cathode peak, is .56. It is known that when the value of b is .5, the electrode material behaves as a cell, and the current is controlled by the diffusive behavior; when the value of b is 1, the current is controlled by a surface-controlled capacitive behavior (Augustyn et al., 2013; Jiang et al., 2017; Li et al., 2018). Therefore, it can be concluded that the SnS@C/G composite exhibits pseudocapacitive behavior. Then, the pseudocapacitive contribution at a given scan rate can be further calculated from Eq. 2.

$$i\left(V\right)/v^{1/2}=k_{1}v^{1/2}+k_{2}$$ (2)

where k_1_ and k_2_ are adjustable parameters and V is the set voltage. At each particular voltage, k_1_ *ν* represents the pseudocapacitive contribution to the current. For the SnS@C/G composite, the pseudocapacitive contribution at 1.0 mV s^−1^ is calculated to be approximately 68.75% as shown in Figure 5C. In addition, the pseudocapacitive contributions at other scan speeds were also calculated as shown in Figure 5D. The capacitance contributions are 42.01, 49.67, 56.88, 61.28, 68.75, and 78.29% at .1, .2, .4, .6, 1.0, and 2.0 mV s^−1^, respectively. The capacitance contribution gradually increases with the increase of scan speed, which demonstrates the predominant role of capacitive storage for SnS@C/G composite during the high-speed discharge/charge.

**FIGURE 6:**
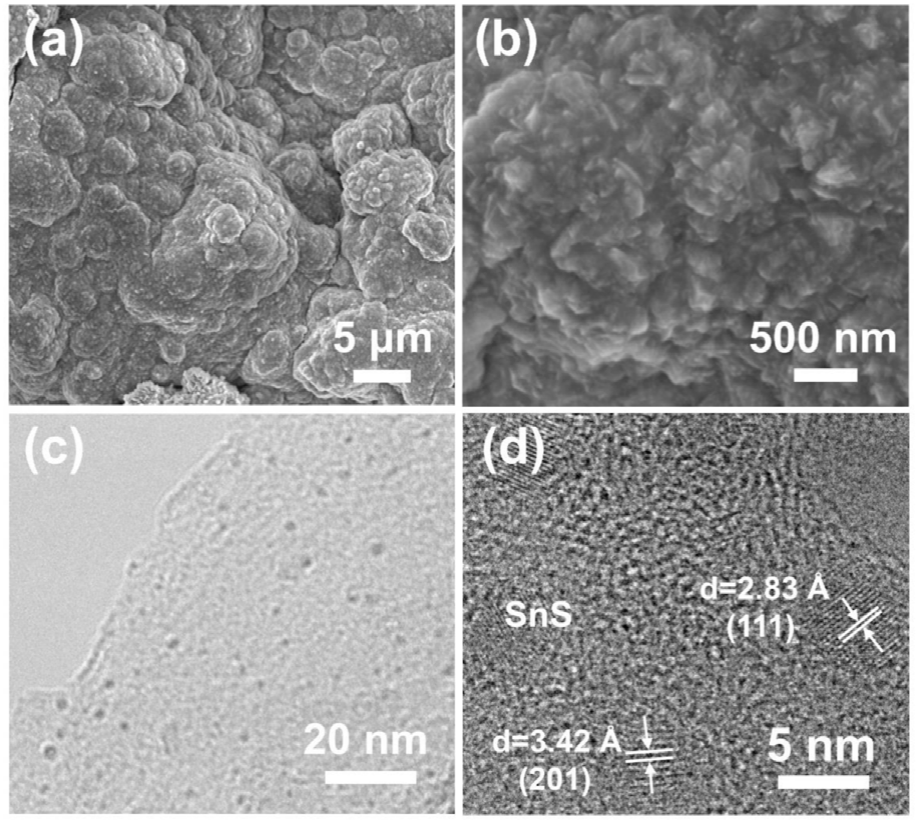
**(A,B)** SEM and **(C,D)** TEM images of SnS@C/G composite after 100 cycles.

Moreover, the morphology of SnS@C/G composites after 100 cycles was observed to further analyze and interpret the outstanding lithium storage performance and behavior of the SnS@C/G composite. As shown in Figures 6A, B of the SEM images of the SnS@C/G composite, there is no obvious sheets-shaped structure can be observed, which is different from the surface morphology of the as-prepared SnS@C/G composite as shown in Figures 2A–C. This indicates the agglomeration of graphene oxide in SnS@C/G composite. To further observe the internal microstructure of the SnS@C/G composite after 100 cycles, the TEM and HRTEM characterizations were further conducted. As presented in Figure 6C, many abundant nanoparticles are still embedded in nanosheets. Furthermore, the HRTEM image shown in Figure 6D still displays the lattice spacings of 3.42 Å and 2.83 Å, corresponding to the (201) and (111) planes of SnS, which are similar to that shown in Figure 2E. Therefore, these results suggest that graphene oxide and N-doped carbon can suppress the SnS aggregation and promote its reversible conversion reaction during repeated lithiation/delithiation.

Based on the above discussion, it can be concluded that the attractive and unique structure of SnS@C/G composites contributes to their outstanding lithium storage performance. Firstly, the ultra-small SnS nanoparticles can provide more active locations for lithium storage and shorten the lithium diffusion length. Secondly, the graphene oxide nanosheets can effectively buffer the volume change of SnS nanoparticles and significantly increase the electrical conductivity to speed up the electrons and lithium ions transport during the discharge/charge process. Thirdly, the N-doped carbon layer can further act as a buffer for the volume change of SnS nanoparticles and also avoid the direct contact between electrolyte and SnS nanoparticles. Finally, the graphene oxide and N-doped carbon can avoid the coarsening of Sn and dissolution of Li_2_S during the discharge/charge process, while provide sufficient electrochemical reaction kinetics to promote the reversibility of conversion reaction of SnS during the cycling.

## 4 Conclusion

In summary, we have successfully designed and synthesized SnS@C/G composites by *in situ* complexation and polymerization reactions followed by *in situ* sulfuration treatment, where SnS@C nanoparticles are strongly coupled onto graphene oxide nanosheets *via* the PDA-derived carbon species. The graphene oxide and N-doped carbon layer in SnS@C/G composites can buffer the volume change of SnS, inhibit the coarsening of Sn, and dissolve Li_2_S in repeated lithiation/delithiation processes. The high conductivity of graphene oxide and N-doped carbon can also increase the transfer rate of electrons and lithium ions during the discharge/charge processes. Therefore, the SnS@C/G composite exhibits outstanding rate performance (434 mAh g^−1^ at a current density of 5.0 A g^−1^) and also excellent cycle stability (839 mAh g^−1^ at 1.0 A g^−1^ after 450 cycles).

## Data Availability

The original contributions presented in the study are included in the article/Supplementary Material, further inquiries can be directed to the corresponding authors.
