# Supplementary material for: SnS@C nanoparticles anchored on graphene oxide as high-performance anode materials for lithium-ion batteries
Source: Front Chem. 2023 Jan 4;10:1105997. doi: 10.3389/fchem.2022.1105997 (PMC9845928; doi:10.3389/fchem.2022.1105997)
Supplement: Supplementary file 1 [file DataSheet1.docx]

Supplementary Material
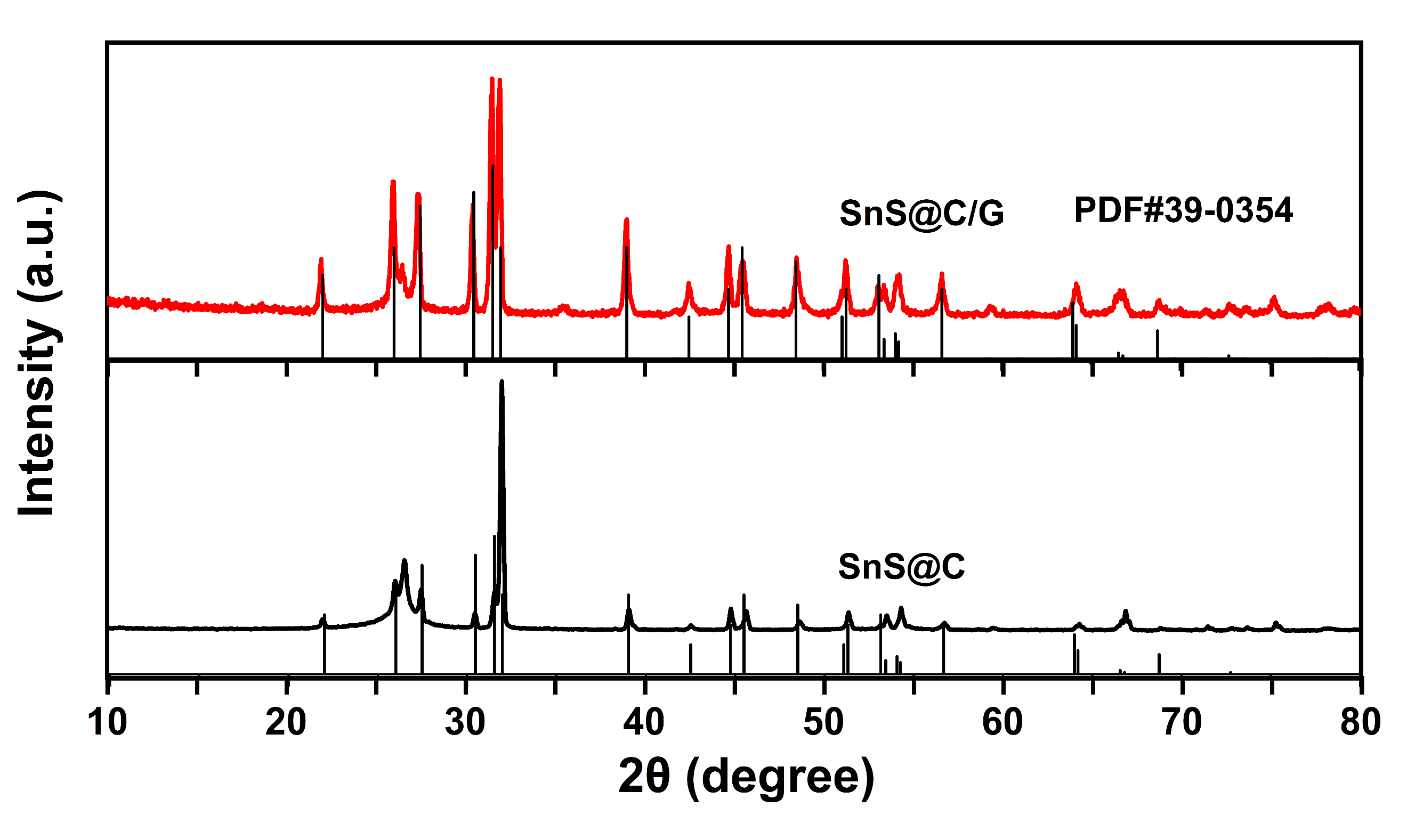


Supplementary Figure 1. XRD patterns of the SnS@C/G and SnS@C composites along with the standard card of SnS (JCPDS No. 39-0354).


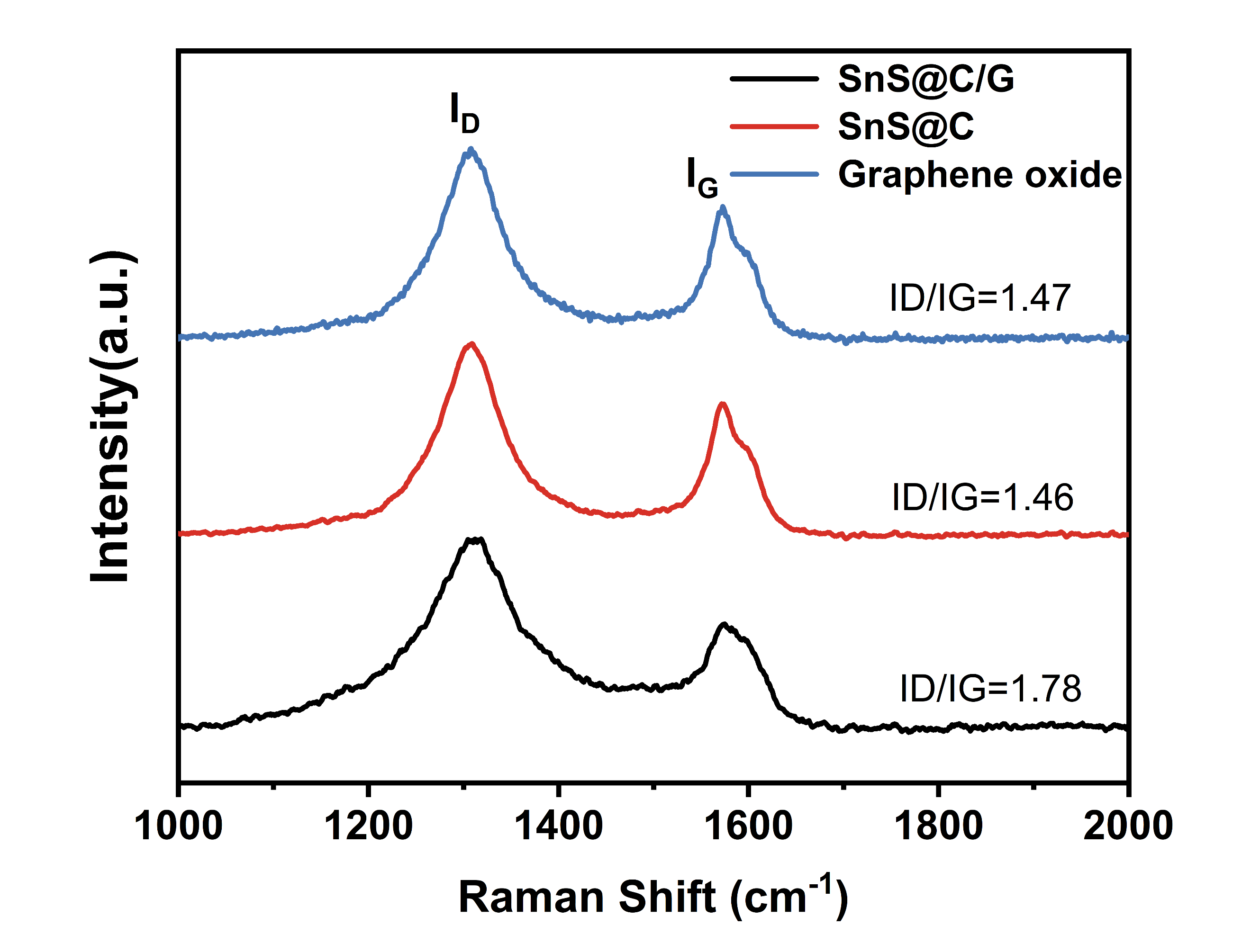


Supplementary Figure 2. Raman spectra of the SnS@C/G composite, SnS@C composite, and graphene oxide.


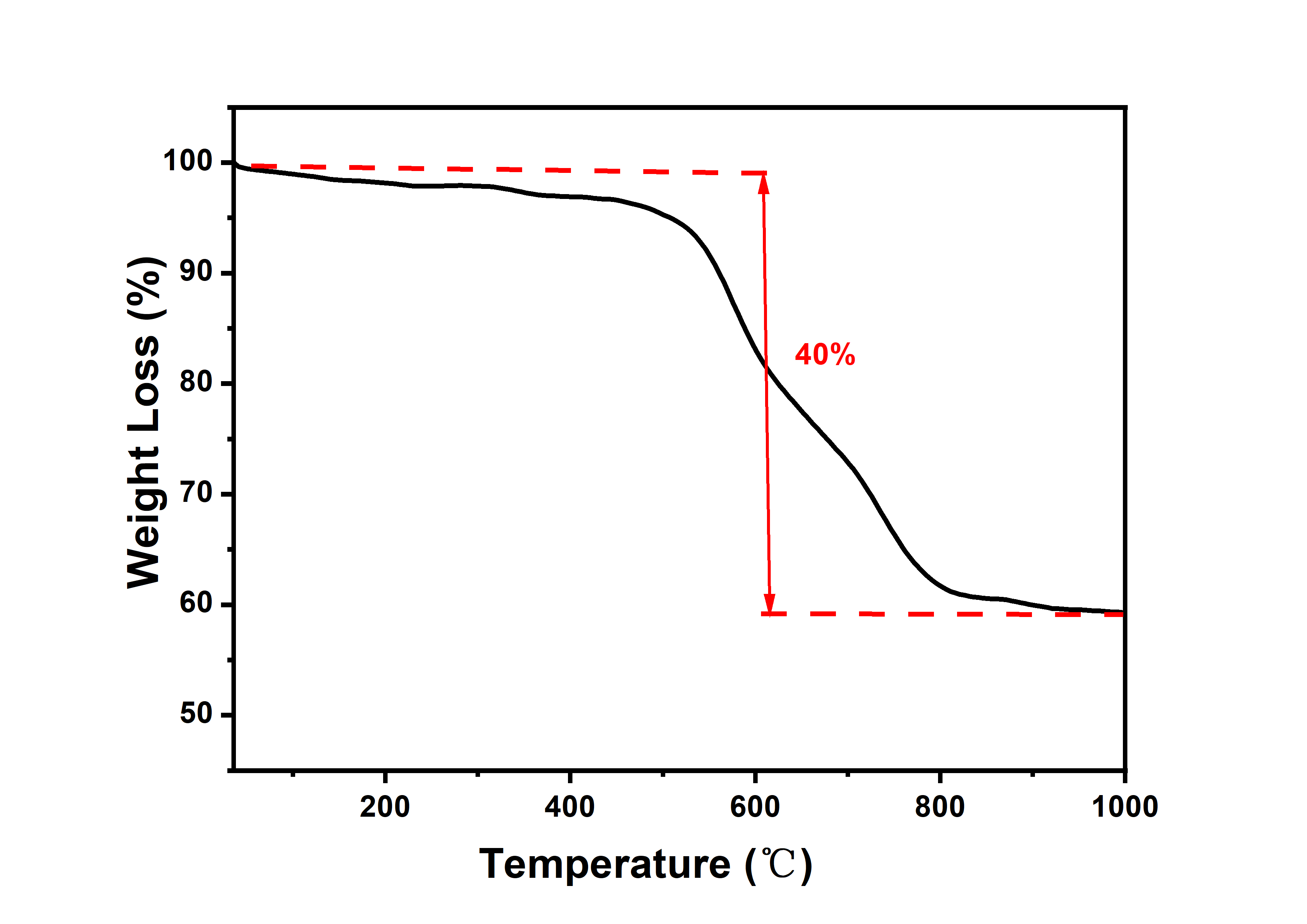


Supplementary Figure 3. TGA curve of the SnS@C/G composite.


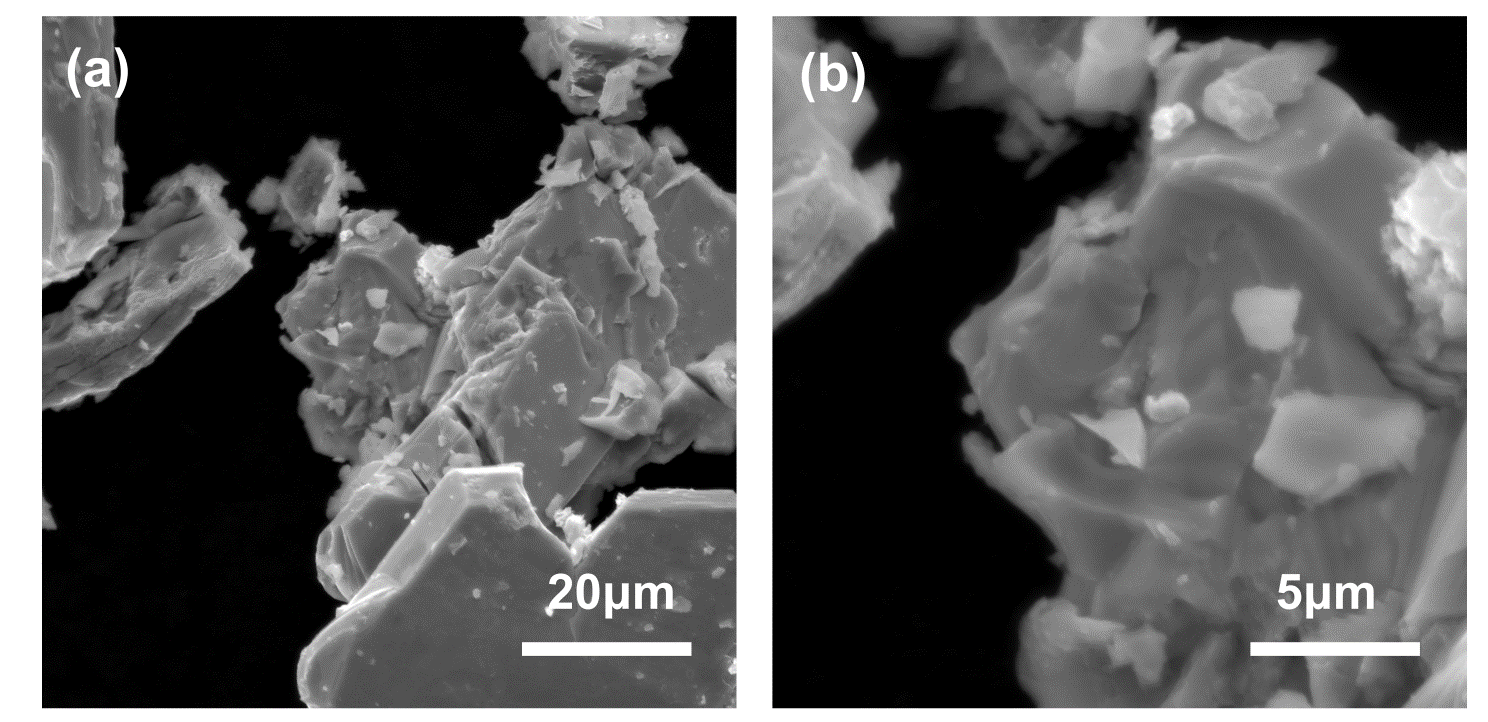


Supplementary Figure 4. SEM images of the SnS@C composite.


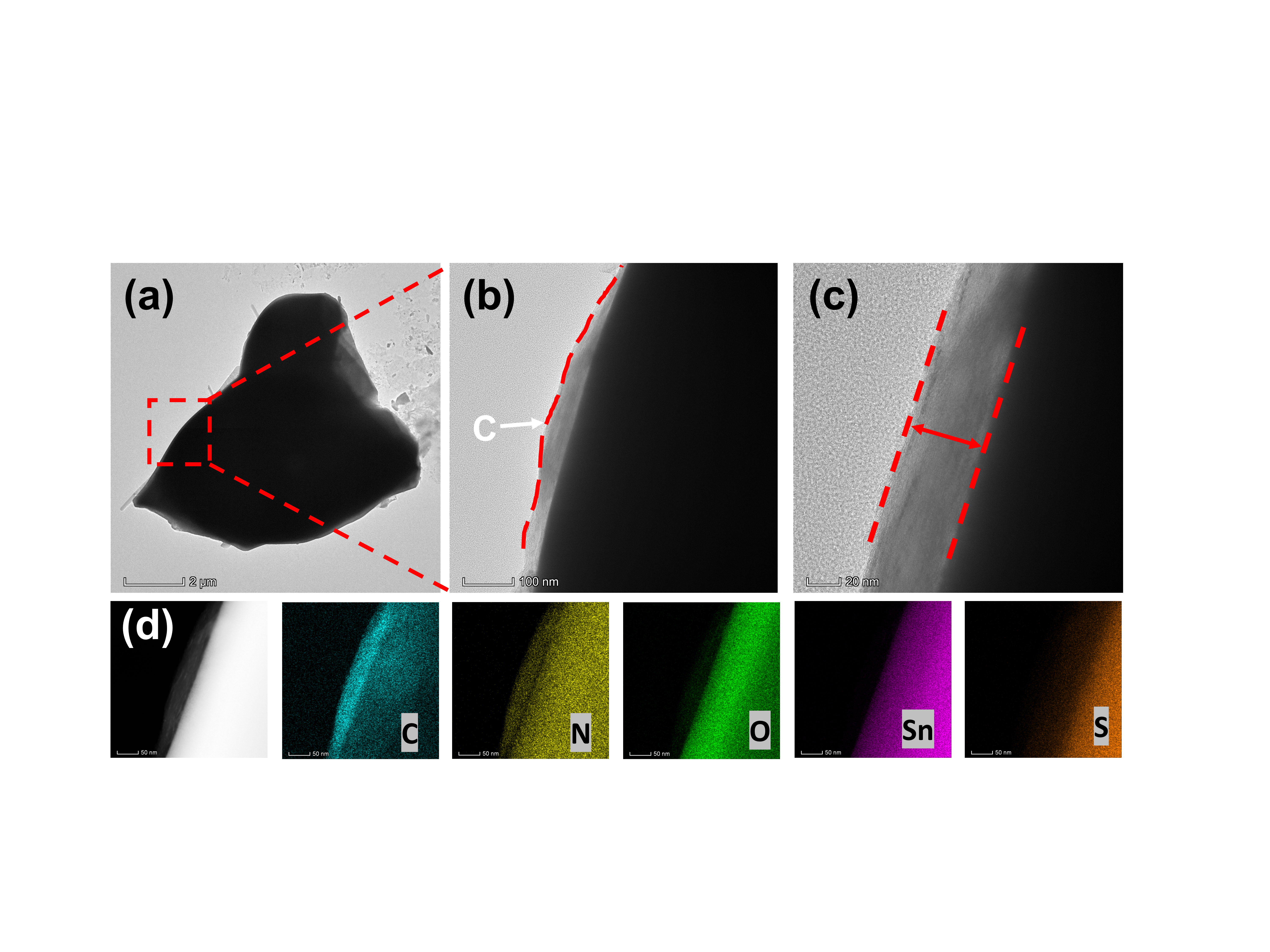


**Supplementary Figure 5.** SnS@C composite (a,b,c) TEM images, (d) STEM image and corresponding elemental mappings of C, N, O, Sn, and S.
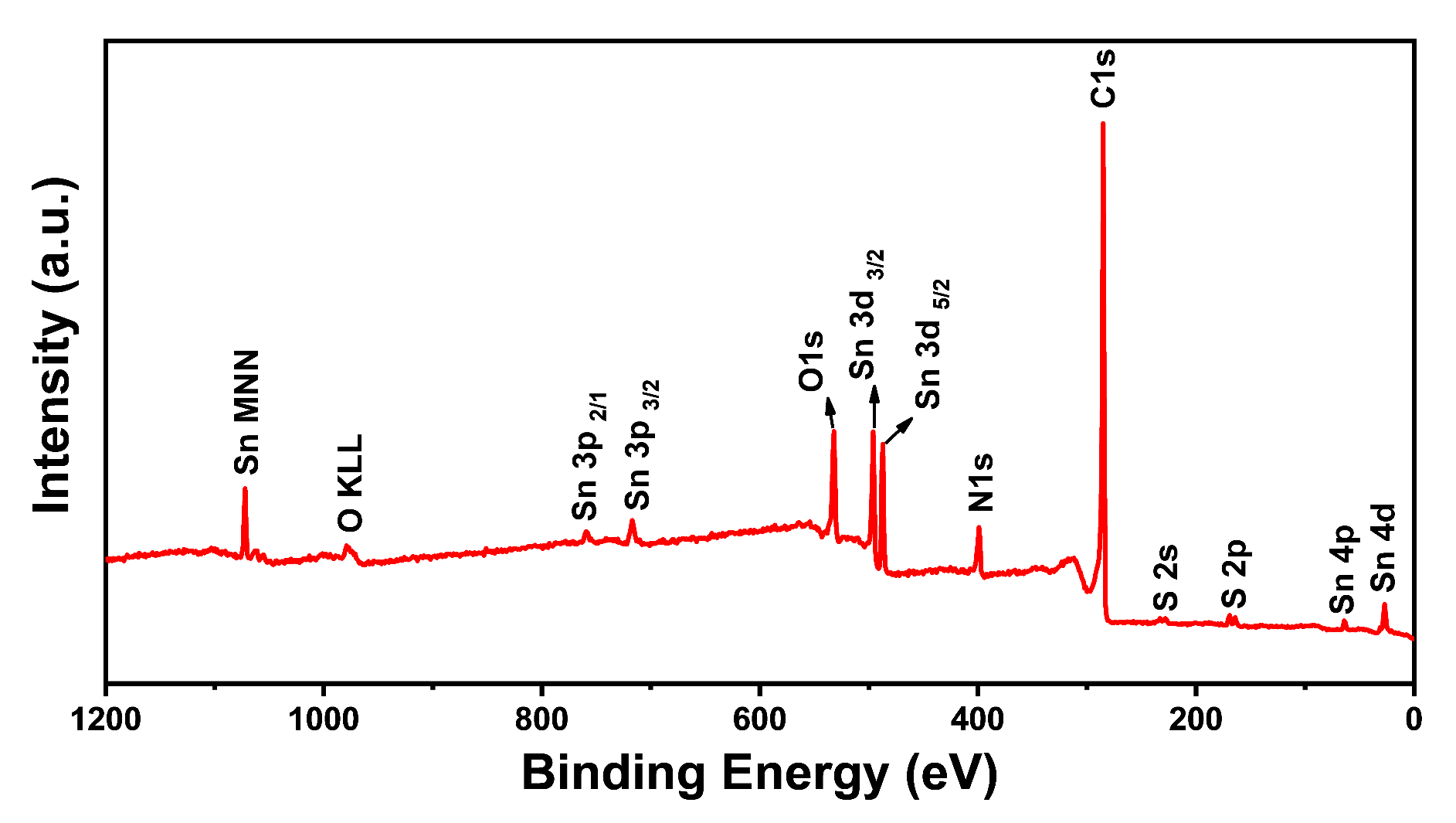


Supplementary Figure 6. XPS survey spectrum of the SnS@C/G composite.


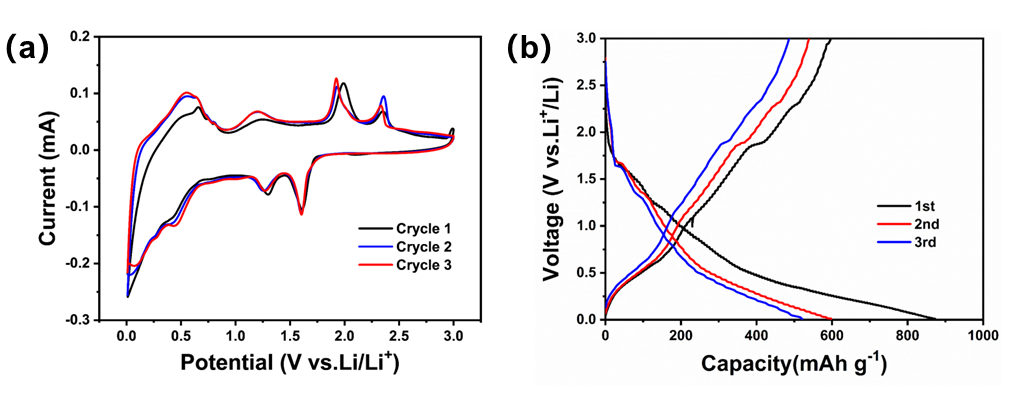


Supplementary Figure 7. (a) CV profiles and (b) charge and discharge proﬁles of the SnS@C composite for the ﬁrst three cycles at 0.1A g^-1^.


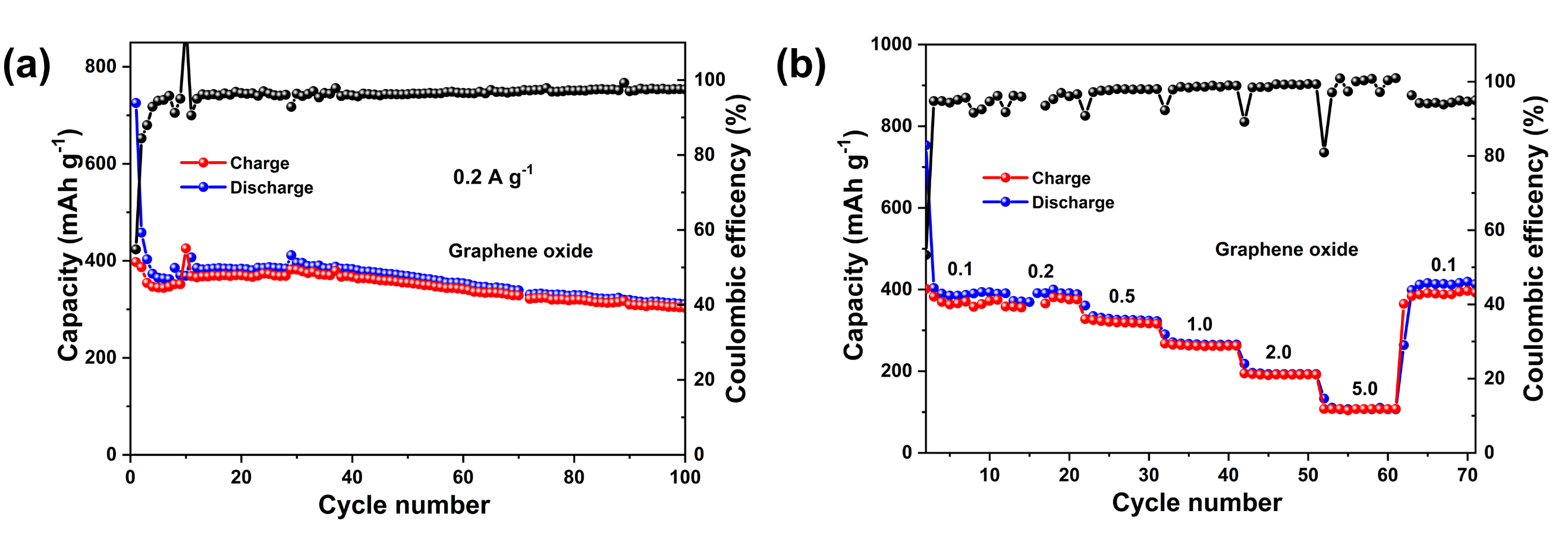


Supplementary Figure 8. Electrochemical performances of the graphene oxide: (a) cycling performance at 0.2 A g^-1^ and (b) rate performance.

Supplementary Table 1. Comparison of specific capacity at different current densities for SnS-based composite anodes reported previously.

| **Materials** | **Capacity retention** | **Rate performance** | **Ref** |
| --- | --- | --- | --- |
| SnS@C/G | 839 mAh g^-1^ after 450 cycles at 1.0 A g^-1^ | 434 mAh g^-1^ at 5.0 A g^-1^ | This Work |
| SnS/RGO | 760 mAh g^-1^ after 100 cycles at 0.1 A g^-1^ | 320 mAh g^-1^ at 1.0 A g^-1^ | (Jin et al., 2016) |
| SnS/S-G | 772 mAh g^-1^ after 100 cycles at 0.1 A g^-1^ | 705 mAh g^-1^ at 1.0 A g^-1^ | (Zhao et al., 2018) |
| SnS/N-doped carbon | 875 mAh g^-1^ after 50 cycles at 0.1 A g^-1^ | 632 mAh g^-1^ at 2.0 A g^-1^ | (Wu et al., 2018) |
| SnS/S-GNS | 894 mAh g^-1^ after 100 cycles at 0.1A g^-1^ | 364 mAh g^-1^ at 10.0 A g^-1^ | (Jiang et al., 2020) |
| SnS–RGO | 613 mAh g^−1^ after 100 cycles at 0.05 A g^-1^ | 198 mAh g^−1^ at 1.0 A g^-1^ | (Choi et al., 2018) |
| NC/SnS/G | 840 mAh g^-1^ after 150 cycles at 0.1 A g^-1^ | 120 mAh g^-1^ at 2.0 A g^-1^ | (Ma et al., 2019) |
| SnS/C NFs-650 °C | 548 mAh g^-1^ after 500 cycles at 0.5 A g^-1^ | 206 mAh g^-1^ at 4.0 A g^-1^ | (Xia et al., 2019) |
| Nanofibrous SnS/C composite | 612 mAh g^-1^ after 70 cycles at 0.1 A g^-1^ | 298 mAh g^-1^ at 1.0 A g^-1^ | (Li et al., 2021) |
| SnS/rGO | 559 mAh g^-1^ after 70 cycles at 0.2 A g^-1^ | 454 mAh g^-1^ at 2.0 A g^-1^ | (Li et al., 2017) |

**
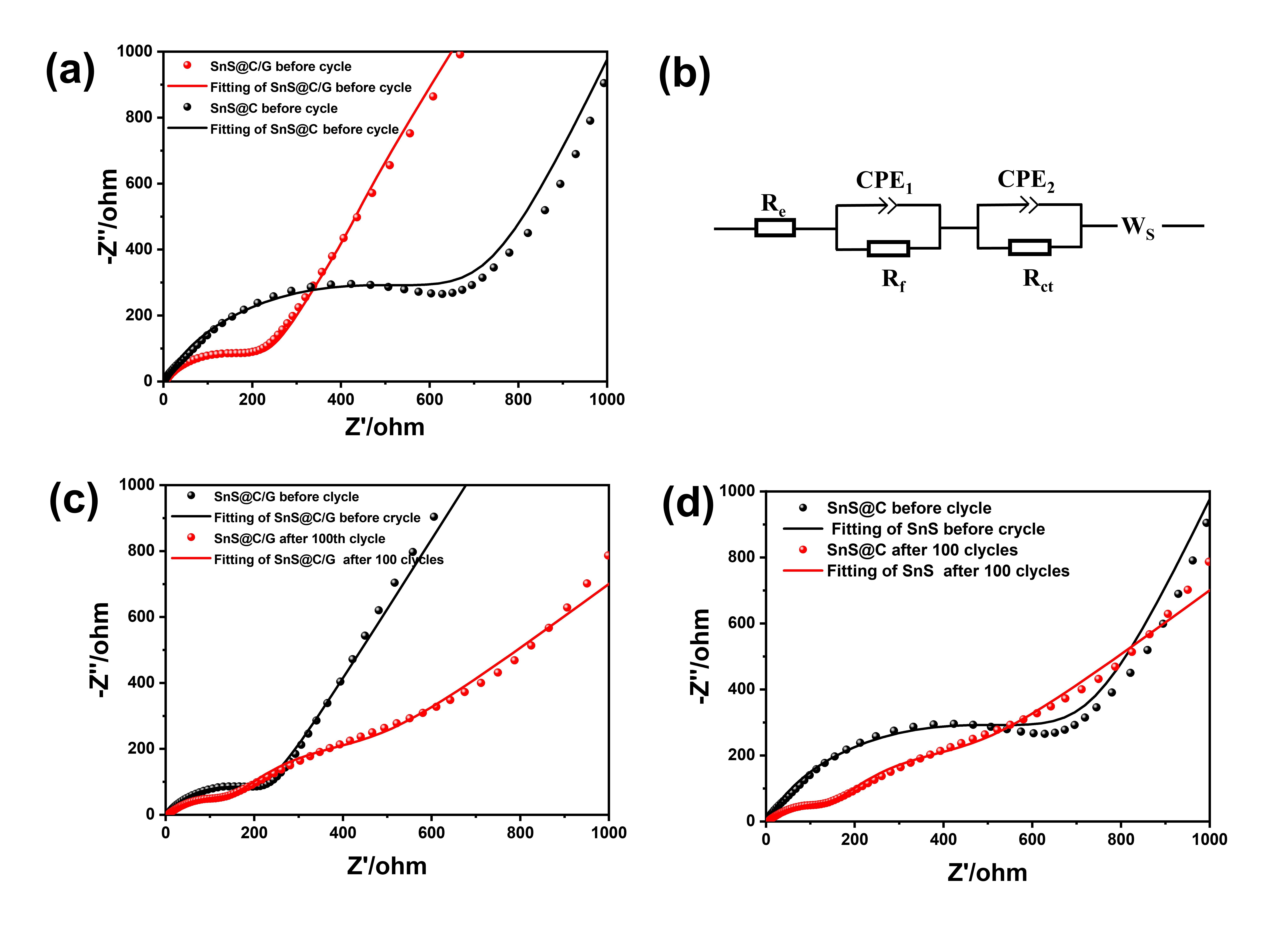
**

Supplementary Figure 9. (a) Nyquist plot measured at the cut-off voltage of 3.0 V in the initial discharge process, (b) the equivalent circuit, (c) Nyquist plots of SnS@C/G and (d) SnS@C before and after 100 cycles.


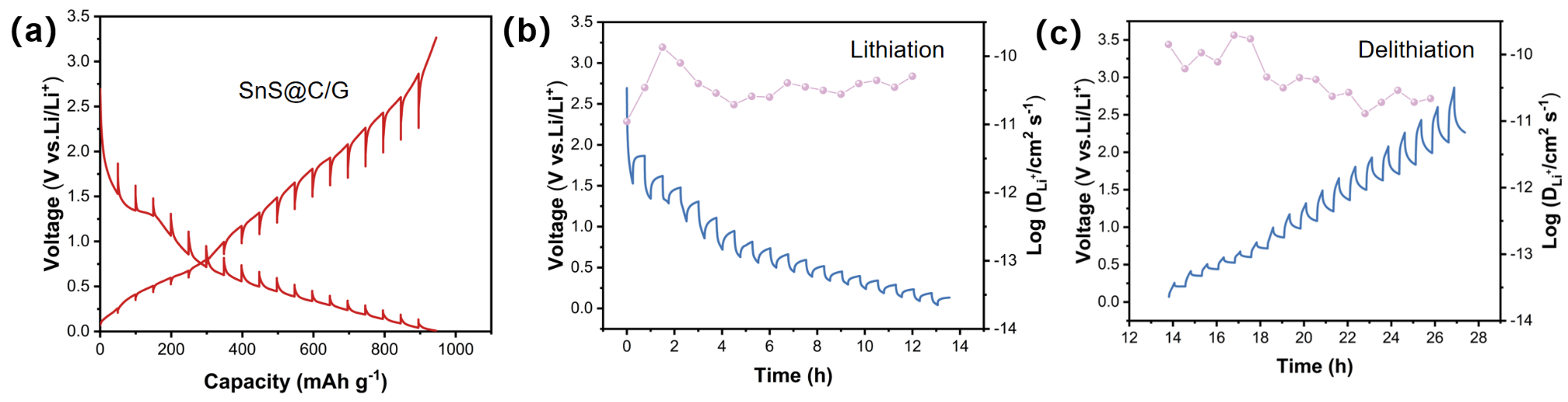


Supplementary Figure 10. GITT potential profiles and calculated Li-ion diffusion coefficients of the SnS@C/G electrode during the (b) lithiation and (c) delithiation processes.

Here, τ is the time of the current pulse. *m_B_*, *V_M_*, and *m_B_* are the mass, molar volume, and molar mass of the electrode material, respectively. *S* is the geometric surface area of the electrode. *ΔEs* and Δ*Eτ* correspond to the steady-state voltage variation during a single GITT titration and the total voltage variation during a current pulse t of a single GITT titration, respectively. Specifically, *m_B_*/*V_M_*, can be obtained from the density of the electrode material. The GITT profile of SnS@C/G indicates a stable discharge process with a *DLi^+^* range of 7.97×10^-11^ to 1.35×10^-10^ cm^2^ s^-1^ for SnS@C/G throughout the charging in lithiation/demineralization process, and a higher diffusion Li^+^ coefficients indicate faster electrochemical reaction kinetics for SnS@C/G electrodes.

**References**

Choi, M., William, W., Hwang, J., Yoon, D., and Kim, J. (2018). A supercritical ethanol route for one-pot synthesis of tin sulfide-reduced graphene oxides and their anode performance for lithium ion batteries. *J. Ind. Eng. Chem.* 59**,** 160-168. doi: 10.1016/j.jiec.2017.10.020.

Jiang, Y., Ding, Y., Chen, F., Wang, Z., Xu, Y., Huang, S., et al. (2020). Structural phase transformation from SnS_2_/reduced graphene oxide to SnS/sulfur-doped graphene and its lithium storage properties. *Nanoscale* 12(3)**,** 1697-1706. doi: 10.1039/c9nr08075a.

Jin, H., Gu, M., Ji, S., Xu, X., and Liu, J. (2016). Reduced graphene oxide anchored tin sulfide hierarchical microspheres with superior Li-ion storage performance. *Ionics* 22(10)**,** 1811-1818. doi: 10.1007/s11581-016-1712-3.

Li, J., Wang, M., and Huang, J. (2021). Bio-inspired hierarchical nanofibrous SnS/C composite with enhanced anodic performances in lithium-ion batteries. *J. Alloys Compd.* 860. doi: 10.1016/j.jallcom.2020.157897.

Li, J., Zhao, X., and Zhang, Z. (2017). Ultradispersed nanoarchitecture of SnS nanoparticles/reduced graphene oxide for enhanced sodium storage performance. *J. Colloid Interface Sci.* 498**,** 153-160. doi: 10.1016/j.jcis.2017.03.056.

Ma, T., Sun, L., Niu, Q., Xu, Y., Zhu, K., Liu, X., et al. (2019). N-doped carbon-coated Tin sulfide/graphene nanocomposite for enhanced lithium storage. *Electrochim. Acta* 300**,** 131-137. doi: 10.1016/j.electacta.2019.01.104.

Wu, C.Y., Yang, H., Wu, C.Y., and Duh, J.G. (2018). Flower-like structure of SnS with N-doped carbon via polymer additive for lithium-ion battery and sodium-ion battery. *J. Alloys Compd.* 750**,** 23-32. doi: 10.1016/j.jallcom.2018.03.386.

Xia, J., Liu, L., Jamil, S., Xie, J., Yan, H., Yuan, Y., et al. (2019). Free-standing SnS/C nanofiber anodes for ultralong cycle-life lithium-ion batteries and sodium-ion batteries. *Energy Stor. Mater* 17**,** 1-11. doi: 10.1016/j.ensm.2018.08.005.

Zhao, B., Yang, Y., Wang, Z., Huang, S., Wang, Y., Wang, S., et al. (2018). In-situ sulfuration synthesis of sandwiched spherical tin sulfide/sulfur-doped graphene composite with ultra-low sulfur content. *J. Power Sources* 378**,** 81-89. doi: 10.1016/j.jpowsour.2017.12.005.
